# Complete chloroplast genome of endangered species *Dipterocarpus retusus* Blume (Dipterocarpaceae) and its phylogenetic implications

**DOI:** 10.1080/23802359.2024.2387257

**Published:** 2024-08-30

**Authors:** Yu-Tian Tao, Meng Yuan, Qin-Qin Li, Zhong-Shuai Sun

**Affiliations:** a School of Electronics and Information Engineering, Taizhou University, Taizhou, China; b Zhejiang Provincial Key Laboratory of Plant Evolutionary Ecology and Conservation, Taizhou University, Taizhou, China; c College of Life Sciences, Shanghai Normal University, Shanghai, China; d College of Life Sciences, Taizhou University, Taizhou, China

**Keywords:** *Dipterocarpus retusus*, *Dipterocarpus*, chloroplast genome, phylogenomics

## Abstract

*Dipterocarpus retusus* Blume is an endangered species on the IUCN Red List. In this study, we reported the complete chloroplast (cp) genome of *D. retusus* (GenBank accession number: OP271853). The cp genome was 154,303 bp long, with a large single-copy (LSC) region of 85,586 bp and a small single-copy (SSC) region of 20,273 bp separated by a pair of inverted repeats (IRs) of 24,222 bp. It encodes 128 genes, including 84 protein-coding genes, 36 tRNA genes, and eight ribosomal RNA genes. We also reconstructed the cp genome phylogeny of *Dipterocarpus*, which indicated *D. retusus* was closely related with the sympatric species *D. gracilis*. This study may contribute valuable information to the phylogenetic relationships within the genus *Dipterocarpus*.

## Introduction

Species of Dipterocarpaceae are regarded as the symbolic species of South-east Asian tropical rain forests and many seasonally dry forests (Brearley et al. 2017). Many species in Dipterocarpaceae are the most important and valuable source in the timber market for producing large quantity and high-quality wood (Schulte and Schöne 1996). The borneol obtained from some Dipterocarpaceae trees has been widely used in the fields of medicine, pesticide, and chemical industry (Yang et al. 2020; Dong et al. 2021). Although Dipterocarpaceae is important to forest ecology, medicine, and conservation, the classifications of Dipterocarpaceae (e.g. the delineation of genera *Parashorea* and *Shorea*) still remain controversial (Cvetković et al. 2019; Zhu and Sun 2019; Yu et al. 2021). Over the past few decades, next-generation sequencing has experienced rapid advancements, providing accurate, comprehensive, and cost-effective approaches for *de novo* assembly of organelle genomes, and the gene-rich plastid genome has been widely used for inferring phylogenetic relationships of closely related plant species (Jin et al. 2020; Tao et al. 2020). *Dipterocarpus retusus* Blume 1868 is an endangered species on the IUCN Red List (Bodos et al. 2019) which has a native range from Assam to China (W. & SE. Yunnan), Indo-China to Lesser Sunda Islands. This species has high economic value as an important timber species and ecological value as a key structural component of the rain forest. Therefore, we assembled and characterized the complete chloroplast (cp) genome of *D. retusus*, with the aim of establishing a solid foundation for future phylogenetic investigations and medical applications of *Dipterocarpus*.

## Materials and methods

**Figure 1.**
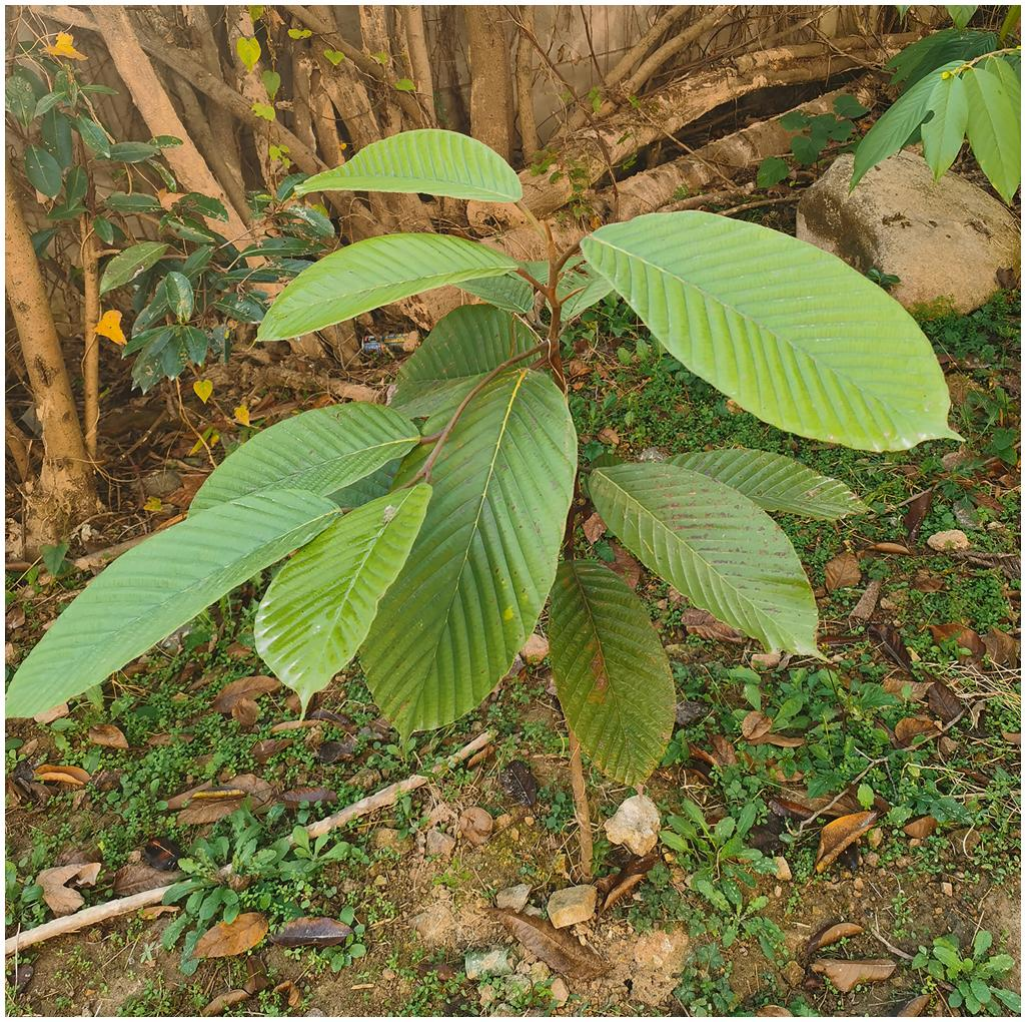
The morphology of *D. retusus*. The leaves of *D. retusus* are broadly ovate in shape, and the petioles are devoid of hair, unlike the same genus species of *Dipterocarpus gracilis* Blume, which exhibits oblong leaves and stellate hairy petioles. Pictures were taken by the author Zhong-Shuai Sun on 2022-06-01 in Linhai campus of Taizhou University that located in Zhejiang province of China.

Leaf material of *D. retusus* (Figure 1) was collected from the Linhai campus of Taizhou University, Linhai, Zhejiang province, China (28.8808N, 121.1722E). A voucher specimen was deposited at Herbarium of Taizhou University (https://www.tzc.edu.cn/; collector: Zhong-Shuai Sun, sun2143998@163.com) under the voucher number Sun2108001. Total genomic DNA was extracted as reported in Chen et al. (2022).

Sequencing was performed with an Illumina NovaSeq platform (Illumina, San Diego, CA). The cp genome was assembled via NOVOPlasty 2.6.3 (Dierckxsens et al. 2017), using the *Dipterocarpus tempehes* (NC026839, Undaharta et al. 2020) as reference. The cp genome annotation was performed online using GeSeq v.1.59 (Tillich et al. 2017) by comparing the cp genome sequence of *D. tempehes* (NC026839, Undaharta et al. 2020). Geneious R11 (Biomatters Ltd., Auckland, New Zealand) was used for inspecting the cp genome structure (Kearse et al. 2012). Circular cp genome map was visualized with CPGView software (Liu et al. 2023).

The phylogenetic relationship with *Dipterocarpus* was evaluated based on an alignment of concatenated protein-coding regions (CDS) among all predicted protein coding genes by the software MAFFT v7.475 (Katoh and Standley 2013). The CDS sequences were extracted from 18 complete cp genomes of Dipterocarpaceae. *Shorea zeylanica* (NC040965, Heckenhauer et al. 2019) and *Parashorea chinensis* (NC046579, Zhu and Sun 2019) were used as outgroups. A maximum-likelihood (ML) analysis was conducted using RAxML-HPC v.8.2.10 on the CIPRES cluster (Miller et al. 2010). GTRCAT model and rapid bootstrap analysis (1000 replicates) were employed for ML inference.

## Results

**Figure 2.**
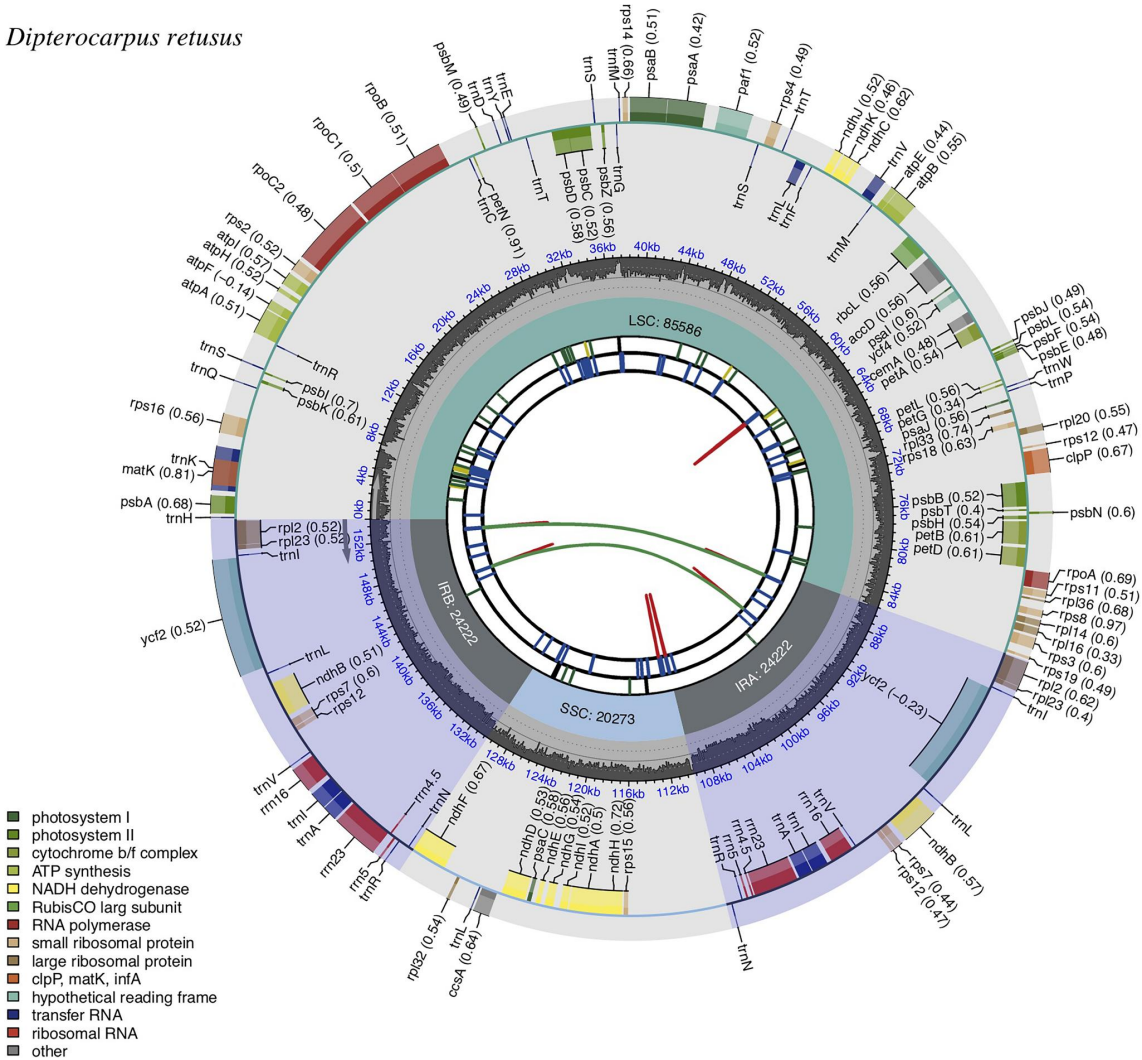
Schematic map of *D. retusus* chloroplast genome constructed by CPGview (http://www.1kmpg.cn/cpgview/). The map contains six tracks in default. From the center outward, the first track shows the dispersed repeats connected with arcs. The second track shows the long tandem repeats as short bars. The third track shows the short tandem repeats or microsatellite sequences as short bars. The small single-copy (SSC), inverted repeat (IRs), and large single-copy (LSC) regions are shown on the fourth track. The GC content along the genome is plotted on the fifth track. The genes are shown on the sixth track. The optional codon usage bias is displayed in the parenthesis after the gene name. Genes are coded by their functional classification. The transcription directions for the inner and outer genes are clockwise and anticlockwise, respectively. The functional classification is shown at the bottom left.

The complete cp genome of *D. retusus* spanned a length of 154,303 bp. The read coverage depth was sufficient (with an average of 1314×), indicating the robustness of genome assembly (Supplementary Figure 1). It exhibited a typical quadripartite structure (Figure 2) which was common in angiosperms: a large single-copy (LSC) region (85,586 bp), a small single-copy (SSC) region (20,273 bp), and a pair of inverted repeats (IRs) region (24,222 bp). The overall GC contents of the total length, LSC, SSC, and IR regions were 37.0%, 34.8%, 31.5%, and 43.1%, respectively.

In total, 128 genes were annotated in the genome, including 84 protein-coding genes (PCGs), 36 tRNA genes, and eight rRNA genes. Among these genes, 13 genes (*clpP*, *ndhA*, *ndhB*, *petB*, *petD*, *rpl2*, *rpoC1*, *rps16*, *trnA-UGC*, *trnI-GAU*, *trnK-UUU*, *trnL-UAA*, and *trnV-UAC*) had one intron and two genes (*rps12* and *pafI*) had two introns. Most genes were single-copy genes, while six PCGs (*ndhB*, *rpl2*, *rpl23*, *rps7*, *rps12*, and *ycf2*), seven tRNA genes (*trnA-UGC*, *trnI-CAU*, *trnI-GAU*, *trnL-CAA*, *trnN-GUU*, *trnR-ACG*, and *trnV-GAC*), and four rRNA genes (*rrn4.5*, *rrn5*, *rrn16*, and *rrn23*) in IR regions were duplicated. The genome contained 11 cis-splicing genes and one trans-splicing gene (Supplementary Figures 2 and 3).

**Figure 3.**
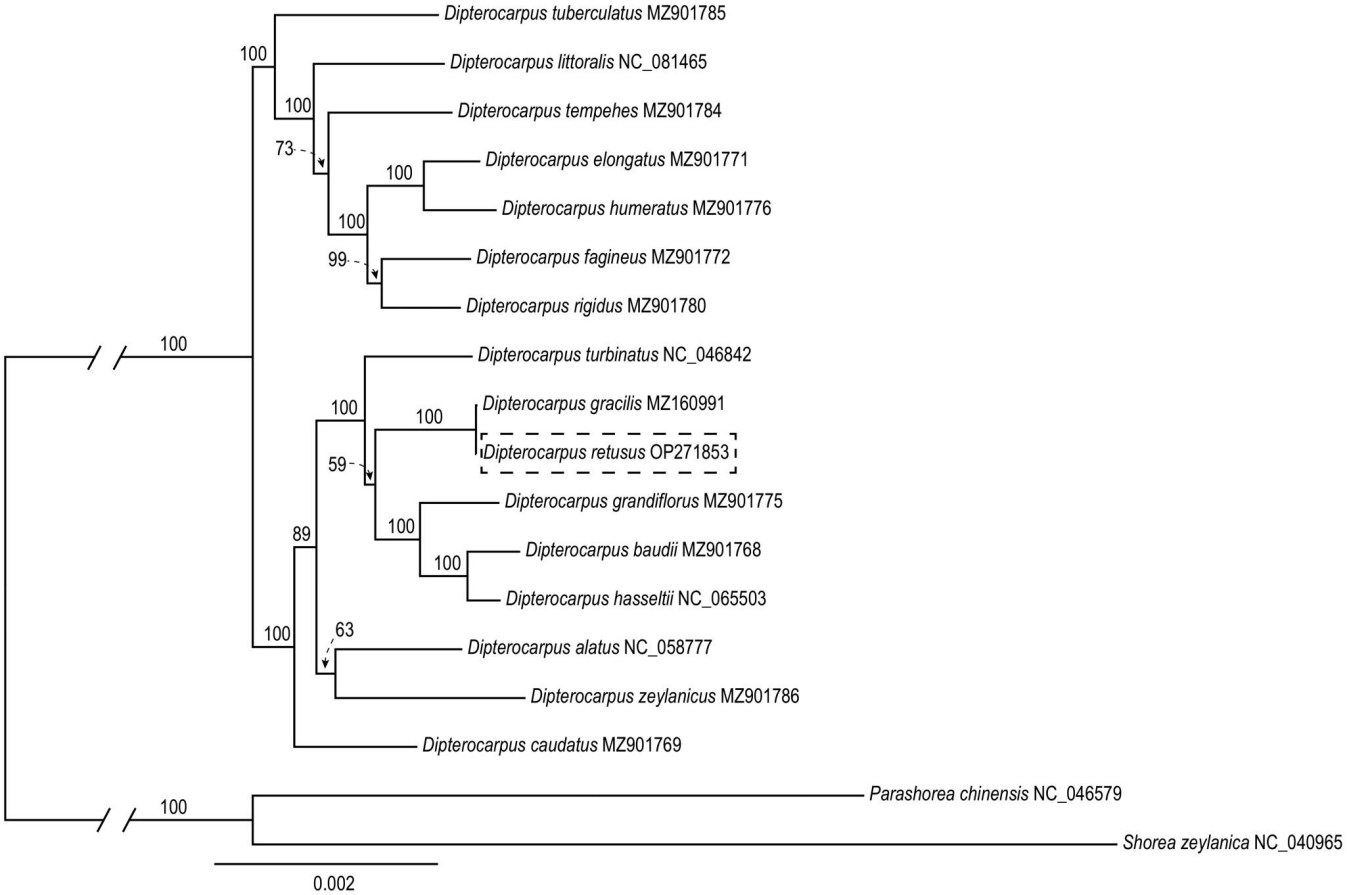
Maximum-likelihood (ML) tree reconstruction of 18 taxa from Dipterocarpaceae based on 84 shared CDS in the chloroplast genomes. Relative branch lengths are indicated. Support values above the branches are ML bootstrap support. The following sequences were used: NC_046579 (Zhu and Sun 2019), NC_040965 (Heckenhauer et al. 2019), MZ160991 (Yu et al. 2021), NC_046842 (Ci et al. 2019), NC_058777 (direct submission to NCBI), NC_065503 (direct submission to NCBI), NC_081465 (direct submission to NCBI), MZ901768 (Cvetković et al. 2022), MZ901769 (Cvetković et al. 2022), MZ901771 (Cvetković et al. 2022), MZ901772 (Cvetković et al. 2022), MZ901775 (Cvetković et al. 2022), MZ901776 (Cvetković et al. 2022), MZ901780 (Cvetković et al. 2022), MZ901784 (Cvetković et al. 2022), MZ901785 (Cvetković et al. 2022), and MZ901786 (Cvetković et al. 2022).

A robust phylogenetic tree of *Dipterocarpus* was obtained based on the CDS data; most nodes in the phylogenetic tree were highly supported (Figure 3). According to the phylogenetic tree, *Dipterocarpus* was resolved as a monophyletic clade consisting two well supported clades. Among the *Dipterocarpus* species analyzed, *D. retusus* was observed to be closely related with *D. gracilis* according to the phylogenetic tree, and the relationship was fully supported.

## Discussion and conclusions

The cp genome of *D. retusus* was assembled using short-read data in this study. It showed that the genome size, GC content, and gene composition of the cp genome sequence of *D. retusus* were similar to those of other species of the genus *Dipterocarpus* (Ci et al. 2019; Yu et al. 2021; Cvetković et al. 2022). Our phylogenetic result coincides with previous molecular phylogeny studies (Jacqueline et al. 2017; Yu et al. 2021), but provides additional insights of the phylogeny of *Dipterocarpus* for involved more *Dipterocarpus* species. Notably, *D. retusus* is closely with a sympatric species *D. gracilis* among the *Dipterocarpus* species analyzed with robust bootstrap support (Figure 3). We expect that the cp genome of *D. retusus* will be a valuable resource for future studies on molecular identification and the better understanding of phylogeny in *Dipterocarpus* and Dipterocarpaceae.

## Supplementary Material

Supplementary Material.docx

## Data Availability

The genome sequence data of this study are openly available in GenBank of NCBI at https://www.ncbi.nlm.nih.gov/ under the accession no. OP271853. The associated BioProject, SRA, and Bio-Sample numbers are PRJNA1054675, SRR27290476, and SAMN38930875, respectively.
