# Supplementary material for: Complete chloroplast genome of endangered species Dipterocarpus retusus Blume (Dipterocarpaceae) and its phylogenetic implications
Source: Mitochondrial DNA B Resour. 2024 Aug 30;9(9):1162–5. doi: 10.1080/23802359.2024.2387257 (PMC11370694; doi:10.1080/23802359.2024.2387257)
Supplement: Supplementary Material.docx [file TMDN_A_2387257_SM4773.docx]

**Supplementary Figure 1** Coverage depth distribution of the *Dipterocarpus retusus* cp genome.


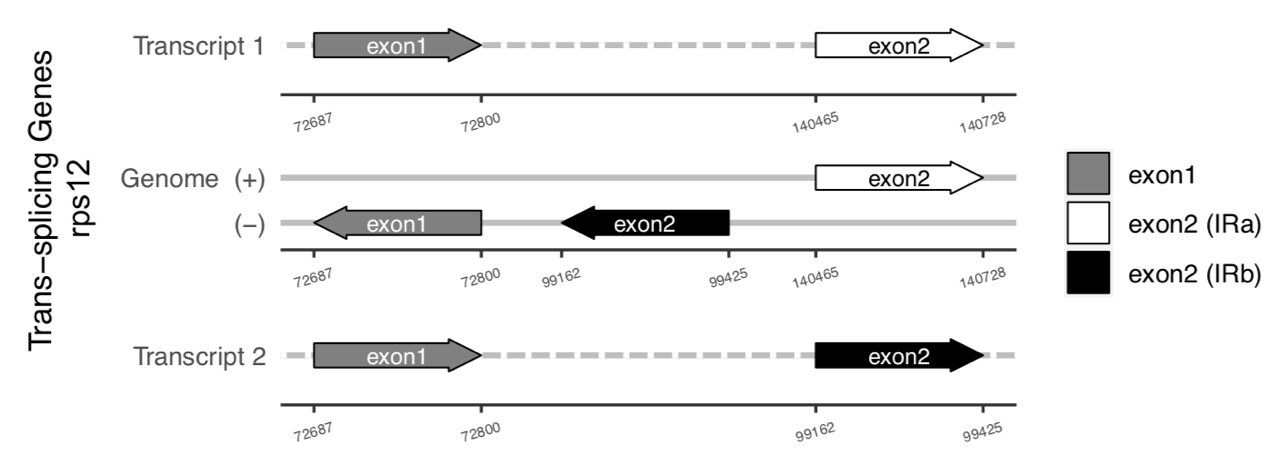


**Supplementary Figure 2**. Structure of trans-splicing genes in the *Dipterocarpus retusus* cp genome.


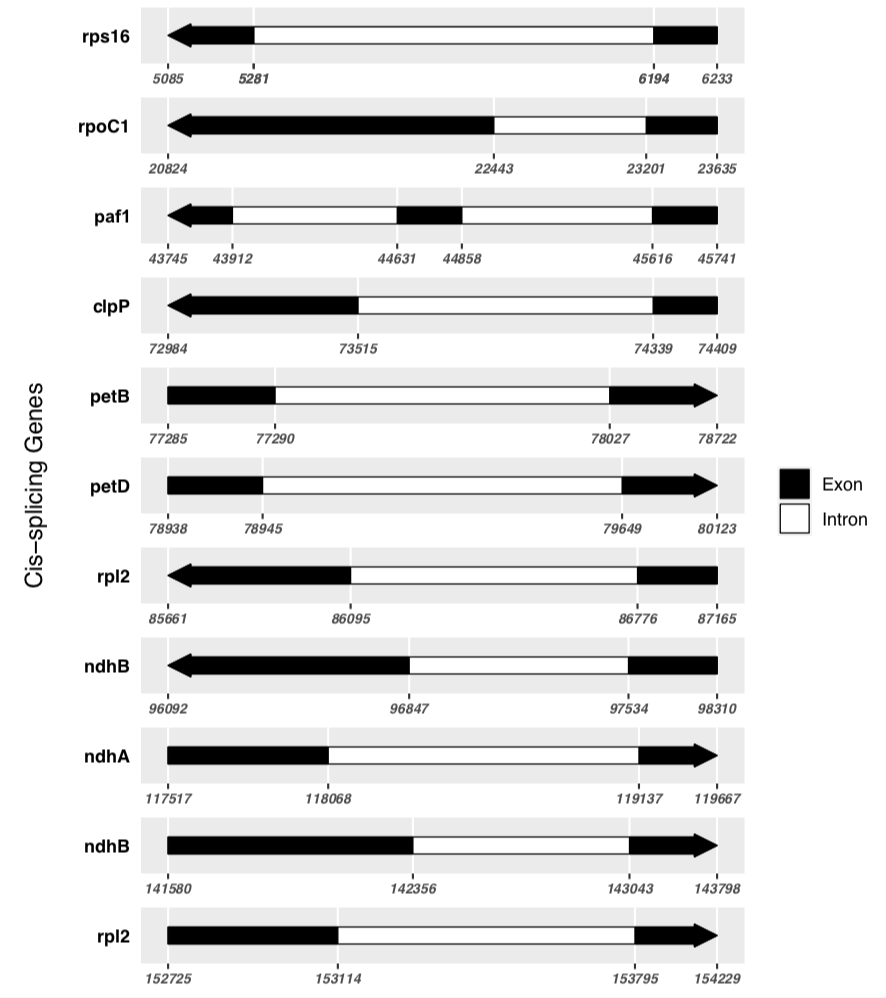


**Supplementary Figure 3** Structure of Cis-splicing genes in the *Dipterocarpus retusus* cp genome.
